# Resampling procedures to identify important SNPs using a consensus approach

**DOI:** 10.1186/1753-6561-5-S9-S59

**Published:** 2011-11-29

**Authors:** Christopher Pardy, Allan Motyer, Susan Wilson

**Affiliations:** 1 Prince of Wales Clinical School, Faculty of Medicine, University of New South Wales, Sydney, New South Wales 2052, Australia; 2 School of Mathematics and Statistics, Faculty of Science, University of New South Wales, Sydney, New South Wales 2052, Australia

## Abstract

Our goal is to identify common single-nucleotide polymorphisms (SNPs) (minor allele frequency > 1%) that add predictive accuracy above that gained by knowledge of easily measured clinical variables. We take an algorithmic approach to predict each phenotypic variable using a combination of phenotypic and genotypic predictors. We perform our procedure on the first simulated replicate and then validate against the others. Our procedure performs well when predicting Q1 but is less successful for the other outcomes. We use resampling procedures where possible to guard against false positives and to improve generalizability. The approach is based on finding a consensus regarding important SNPs by applying random forests and the least absolute shrinkage and selection operator (LASSO) on multiple subsamples. Random forests are used first to discard unimportant predictors, narrowing our focus to roughly 100 important SNPs. A cross-validation LASSO is then used to further select variables. We combine these procedures to guarantee that cross-validation can be used to choose a shrinkage parameter for the LASSO. If the clinical variables were unavailable, this prefiltering step would be essential. We perform the SNP-based analyses simultaneously rather than one at a time to estimate SNP effects in the presence of other causal variants. We analyzed the first simulated replicate of Genetic Analysis Workshop 17 without knowledge of the true model. Post-conference knowledge of the simulation parameters allowed us to investigate the limitations of our approach. We found that many of the false positives we identified were substantially correlated with genuine causal SNPs.

## Background

Our goal is to identify single-nucleotide polymorphisms (SNPs) that add predictive information for the phenotypic outcomes above that given by just the other phenotypes. This aim is motivated by the use of genetic testing in a clinical setting, where SNP genotypes can be used to identify a patient’s risk level better than easily measured clinical variables alone [1].

Our approach combines several well-known statistical procedures: stability selection, random forests, the least absolute shrinkage and selection operator (LASSO), and logistic regression. Random forests are an algorithmic machine learning technique based on a majority vote among a number of randomly varying trees [2,3]. The algorithm generally gives good predictive accuracy at the expense of interpretability. It has the useful property of providing an importance score for each variable determined by how worse prediction becomes when the given variable is removed from the analysis. This score allows us to add an additional filtering step at the outset to remove SNPs that do not provide useful predictive information. The importance score is insensitive to correlation or colinearity between variables (for example, see section 11.1 of Breiman [4]), allowing us to ignore linkage effects until a smaller set of variables is under consideration. Because of its good computational speed, we use the Random Jungle software, which was developed for a previous Genetic Analysis Workshop [3,5,6].

The LASSO procedure is a well-regarded approach to variable shrinkage and selection [3,7,8]. Although the required tuning parameter can be chosen by cross validation [9], in our experience too many uninformative variables can result in the lack of a global minimum deviance, thus making this parameter difficult to use. Our initial use of random forests minimizes this problem.

Multicolinearity causes regression models to fail, so we follow the example set by the authors of the PLINK software [5] and filter data based on variance inflation factors (VIFs). Population substructure can also be an issue [10], and we briefly investigated this using principal components analysis.

To help protect against false positives and improve the generalizability of results, we use a consensus approach involving multiple subsamples. Subsamples (without replacement) from a single trial may give estimates with improved stability [11]. It has been suggested that samples of size *n*/2 perform well [11].

The true simulation model is described by Blangero et al. [12]. An overview of the use of machine learning methods in genetic epidemiology is given by Dasgupta et al. [3].

## Methods

### Combination of approaches

**Figure 1:**
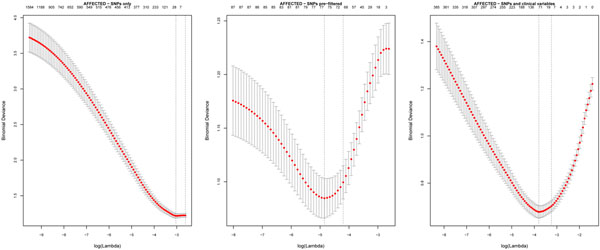
**LASSO cross-validation plots for affected status**. Cross validation fails to identify an appropriate shrinkage parameter by using just the 4,755 SNPs (left-hand panel). A parameter can be chosen when there is additional adjustment for clinical variables (center panel) or when SNPs are prefiltered according to random forest importance score.

When preliminary analyses were confined to the SNPs only as predictors, we found that the LASSO procedure was unable to find a global minimum cross-validation error to select the shrinkage parameter (left-hand panel of Figure 1). Our use of random forests to prefilter the SNPs was driven by the need to find a minimum error (center panel of Figure 1). Alternatively, adjustment for clinical variables also achieved this (right-hand panel of Figure 1). We applied the random forest importance scores first because this procedure does not exclude multiple correlated variables. Because the LASSO assumes independent predictors, we removed highly correlated variables by means of VIF filtering.

### Procedure

We removed SNPs that had a minor allele frequency (MAF) less than 0.01 or that failed a Hardy-Weinberg equilibrium test, leaving 4,755 SNPs for analysis. Random samples of size 348 were taken without replacement from the 679 subjects in the first replicate of the Genetic Analysis Workshop 17 (GAW17) data set. This procedure was repeated 10 times, with subsequent analyses performed on each subsample. We used a principal components analysis to investigate possible population structure within the SNPs and identified three clear groups that corresponded almost exactly with the three major ethnic groups of the subjects (African, Asian, and European). Many analyses were performed both with and without adjusting for ethnicity to determine whether this was an important confounder. In general, we found that ethnicity effects disappeared once the SNPs were taken into account (interestingly, a random forest fitted to the SNP data could perfectly predict these ethnicity groups).

**Figure 2:**
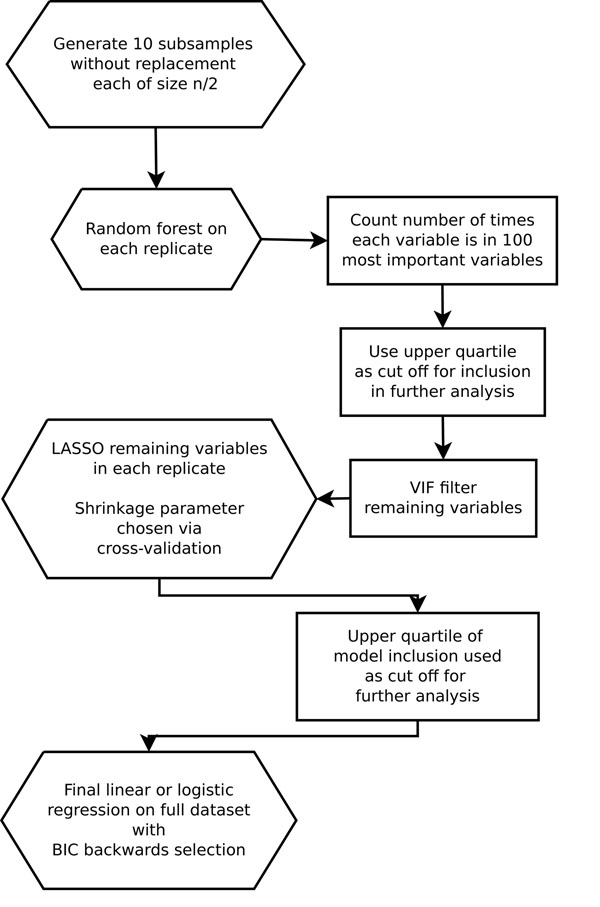
Outline of our approach

We applied the following procedure to each of the subsamples (see Figure 2): (1) We used the Random Jungle program to perform a random forest analysis with 1,000 trees and a sample of 1,000 variables at each node. We assessed variable importance using the gene identification by NMD (nonsense mediated decay) inhibition (GINI) index, which largely matched other calculated importance indexes. (2) We counted the number of times each variable appeared in the 100 most important variables. (3) We chose a cutoff to reduce the set of variables, guided by the upper quartile of inclusion counts. A cutoff resulting in a set of roughly 100 SNPs was found to work well for subsequent stages. (4) Variables were iteratively dropped until none had a VIF greater than 10 when regressed on the others. Because of the lack of substantial pairwise correlation between SNPs, few variables were dropped at this stage. (5) Within each replicate, we used cross validation to choose an appropriate penalty factor for the LASSO. This was done with the cv.glmnet() function in the glmnet R package [9]. The “minimum MSE + 1 standard error” rule was most frequently found to lead to models that were sufficiently sparse to not obviously overfit the data. The random forest filtering step was necessary to ensure a global minimum model-fitting error in the cross-validation step. (6) We counted the number of times each variable was included in each LASSO model (i.e., the number of times each variable had a nonzero estimated coefficient). (7) We chose another cutoff. Once again, the upper quartile was found to be a good choice. (8) Finally, we fitted an unpenalized linear or logistic regression model using all 697 subjects. We used Bayesian information criterion (BIC) backwards selection to remove variables that did not contribute to the model fit. The LASSO step ensured that the maximal model at this stage (before backwards selection) had nonzero deviance and minimized the chance that fitted probabilities were 0 or 1.

We assessed the predictive accuracy over the 200 replicates of the GAW17 data set using the mean-square error (for continuous variables) or the proportion of incorrect predictions (for affected status). Models were fitted both with and without the SNPs to assess whether their inclusion improved accuracy.

## Results

### Predicting Q1

By far the most successful application of our procedure was the prediction of Q1. We observed how frequently SNPs were among the 100 most important variables identified by the random forest. Only 2 SNPs appeared in all 10 subsamples (C13S522 and C13S523). Keeping variables that appeared at least three times (the upper quartile of this distribution) left 90 remaining SNPs. Checking the VIFs caused one SNP to be dropped to avoid colinearity. Similarly, we observed how frequently each variable remained in the model after a subsequent LASSO selection. The upper quartile of this distribution was four, which was used as a cutoff for inclusion in the final model.

We fitted a linear regression using the remaining variables. After BIC backwards selection, we arrived at the “Model with SNPs” in Table 1. These fitted coefficients were used to predict Q1 in each of the other replicates. This gave a set of 199 mean-square prediction errors over the remaining replicates (including the first replicate). We used the median of these as a robust indication of model performance: 0.6899. We refitted the model without the SNPs and similarly validated it, finding a median mean-square error of 0.9868 (Table 1, “Model without SNPs”). This demonstrates that a substantial reduction in prediction error can result from including the identified SNPs, suggesting that we found a set of SNPs with good predictive value.

**Table 1:** Final consensus models for Q1 with and without SNPs

	Estimate	Standard error	p-value
Model with SNPs			
Intercept	−1.45	0.11	<2 × 10^−16^
C10S4601	−0.24	0.10	0.020
C10S4927	0.10	0.04	0.020
C12S2798	−0.07	0.05	0.158
C13S431*	0.45	0.15	0.004
C13S522*	0.80	0.13	1.39 × 10^−9^
C13S523*	0.64	0.09	9.52 × 10^−12^
C14S2902	0.15	0.05	0.002
C18S794	0.14	0.05	0.004
C19S5879	0.11	0.04	0.006
C1S4244	−0.37	0.16	0.018
C1S7427	0.10	0.04	0.013
C4S1220	0.07	0.04	0.081
C5S221	0.11	0.05	0.23
C6S4003	−0.24	0.13	0.057
C6S469	0.30	0.14	0.031
C7S2893	0.09	0.05	0.062
C8S2699	−0.20	0.13	0.114
C9S13	0.13	0.08	0.111
Q2	0.26	0.03	<2 × 10^−16^
Age	0.02	0.00	<2 × 10^−16^

Model without SNPs			
Intercept	−0.93	0.08	<2 × 10^−16^
Q2	0.27	0.03	2.94 × 10^−15^
Age	0.02	0.00	<2 × 10^−16^
Smoke	0.59	0.08	1.01 × 10^−14^

Asterisks indicate a genuine causal variant.

### Post-conference comparisons

Because Q1 was the most amenable to prediction, we decided to use this trait to compare various approaches in light of the true simulated model. To assess the effect of false positives, we fitted a model using only the three SNPs on chromosome 13 known to be casual (with Q1, Age, and Smoke) and found a median mean-square error of 0.6460 over the replicates. In addition, we compared these three SNPs to those SNPs chosen by a simple one-SNP-at-a-time genome-wide association approach with Bonferroni correction. The chosen SNPs were C12S707, C12S711, C12S2028, C12S2798, C13S522, and C13S523 (the chromosome 13 SNPs are genuinely causal). This model had a median mean-square error of 0.6651, with a slight performance improvement over our consensus approach.

### Predicting Q2, Q4, and affected status

The analysis to predict the traits Q2, Q4, and affected status was less successful. Although our procedure was motivated by the attempted analysis of affected status, we were unable to find a model that substantially reduced prediction error. This was also the case for Q2 and Q4, although we did identify a true causal SNP for Q2 (C6S5449).

## Discussion and conclusions

We found that SNPs added predictive information only when Q1 was used as the outcome. Removing SNPs with a low MAF left Q1 as the only outcome that had common enough variants with large enough effect sizes for our approach to be successful. Many of the effect sizes seen in the true simulated model were so small as to be often overshadowed by spurious associations (evidenced by the noncausal SNPs with smaller *p*-values than genuine causal ones).

It is interesting to note that only 15 of the true causal SNPs were included in our analysis after MAF filtering, and only 4 of these were in the top 100 important SNPs from the random forest plot (predicting Q1). These four SNPs correspond to the four correctly identified SNPs. Although one SNP is in the model to predict Q2, this variable is itself associated with Q1 (with an observed correlation of 0.24 in the first replicate). All true identified SNPs had a MAF less than 3%. The choice of using 1,000 variables at each node in the random forest was confirmed by a separate cross-validation study. With hindsight it appears that this was required to ensure that the SNPs with strong effect (on chromosome 13) were selected often enough to reduce prediction error.

We had hoped that the consistent use of resampling-based procedures would stop overfitting of the models on the first replicate. This worked to the extent that prediction accuracy did not become substantially worse by including SNPs for any of the outcomes. The use of subsampling was preferred over simple cross validation because it gave a larger sample size (*n*/2) for each training set with the ability to increase stability by taking more subsamples. However, it would be preferable to have objective criteria for deciding whether a variable should be included at each stage rather than accepting the choices we were forced to make based on computational tractability.

To explain some of the false positives, we calculated correlations between genuine causal SNPs for the consensus model and the naive genome-wide association study (Table 2). Many SNPs identified in our analyses had substantial observed correlations (using our linear SNP coding) greater than 0.2, nearly always across chromosomes. The correlation between SNPs on chromosome 12 and those on chromosome 4 (causal for Q1) potentially explain the cluster of false positives picked up by the genome-wide association study. Many SNPs picked up by the consensus approach were correlated with causal SNPs. The multiple-SNP analysis of our consensus approach minimized this to a reasonable degree, identifying only a single SNP on chromosome 12. Because of our success in predicting Q1, we cannot conclude that the use of random forests as a prefiltering step is completely without merit. If the clinical variables were unavailable, this approach would allow the LASSO model to be fitted.

**Table 2:** Strongest correlations between false positives and genuine causal SNPs

Outcome	Identified SNP	Causal SNP	Correlation
Consensus models^a^			
Q1	C9S13	C4S1878	0.21
	C9S13	C4S1884	0.23
	C1S7427	C6S5380	0.22
	C12S2798	C6S5380	−0.28
	C8S2699	C6S5426	0.21
	C1S7427	C13S523	0.21
	C12S2798	C13S523	−0.27
	C12S7427	C14S3706	−0.27
Q4	C10S6324	C13S523	0.23

Affected	C19S3379	C4S1878	−0.21
	C18S2310	C6S5426	0.26
	C19S3379	C6S5426	0.22
GWAS for Q1^b^	C12S707	C4S1878	0.27
	C12S711	C4S1878	0.28
	C12S707	C4S1884	0.26
	C12S707	C6S5380	0.21
	C12S711	C6S5380	0.21
	C12S2798	C6S5380	−0.28
	C12S707	C13S522	0.21
	C12S711	C13S522	0.21
	C12S707	C13S523	0.45
	C12S711	C13S523	0.4
	C12S2028	C13S523	0.32
	C12S2798	C13S523	−0.27
	C12S707	C14S1734	0.31
	C12S711	C14S1734	0.26
	C12S2028	C14S1734	0.25
	C12S707	C18S2492	0.41
	C12S711	C18S2492	0.26

^a^ SNPs identified by our procedure.

^b^ SNPs identified by a naïve genome-wide association study for Q1.

## Competing interests

The authors declare that there are no competing interests.

## Authors’ contributions

CP carried out the analysis and writing of the paper under the guidance of SW. AM provided the filtered dataset and helpful suggestions.
